# Comment on: Prompt Detection of Intravascular Migration of Cervical Epidural Catheter in Superior Vena Cava Avoids Impending Disaster!

**DOI:** 10.5152/TJAR.2022.213272

**Published:** 2022-10-01

**Authors:** B. Malaka Munasinghe, U. Pasindu M. Fernando

**Affiliations:** 1 Department of Anaesthesiology and Intensive Care, District General Hospital, Mannar, Sri Lanka; 2 National Blood Transfusion Service, Sri Lanka

Dear Editor,

We read the paper titled “Prompt Detection of Intravascular Migration of Cervical Epidural Catheter in Superior Vena Cava Avoids Impending Disaster” published in volume 49, issue 2 of the *Turkish Journal of Anaesthesiology *&* Reanimation*,^1^ with great interest. While it is an insightful read with useful facts to all categories of anaesthetists, we have made the following observations with probable explanations where appropriate.

Thoracic epidural (TE) is considered as the gold standard for provision of analgesia to the patients with multiple rib fractures,^2^ despite recent evidence of unaltered mortality and duration of hospital stay.^3^ Thus, the authors’ preference is justifiable. The failure of placement of the TE may be due to the difficulty in proper positioning (with multiple rib fractures and an in situ intercostal tube) or normal variations in anatomy. It is not clear whether existing vertebral injuries were excluded by the computed tomography scan which could have contributed to the failed attempts. The authors subsequently have opted for a cervical epidural with the catheter threaded in a caudal direction despite the documented risks of dural puncture and cord injury, bilateral intercostal paralysis, worsening respiratory, and haemodynamic parameters related to the procedure. An alternative could have been the delineation of thoracic epidural anatomy by either the ultrasound or fluoroscopy as both modalities were available in the particular centre or choosing bilateral erector spinae blocks (and catheters for continuous infusions), which was successively executed later. In our centre, for similar patients with multiple rib fractures where these regional analgesic modalities are not feasible, we perform serratus anterior plane blocks under ultrasound guidance if the fractures are anterior, or intravenous lignocaine infusions with close monitoring, supplemented with titrated systemic analgesics.

It is quite unusual to have negative aspiration to blood in this particular case with the catheter tip in superior vena cava as multiorifice catheters yield a higher positive results during aspiration to blood, as correctly suggested by the authors. A possible explanation is blockade by a blood clot or tissue fragments at the orifices on initial advancement leading to false-negative aspiration. The patient positioning for cervical epidural may have led to kinking of the catheter, specially if the neck was flexed during advancement of the catheter, later extended during aspiration. Rather than using air, we prefer using saline for loss of resistance technique in our centre due to the familiarity of use and concerns of neurological sequel with the former.^4^

Finally, we would like to acknowledge the authors for reporting this experience which promoted the discussion of formulating a case-based approach in carrying out anaesthetic procedures for the safe provision of epidural analgesia.

## Author’s Reply

**Re: Comment on: Prompt Detection of Intravascular Migration of Cervical Epidural Catheter in Superior Vena Cava Avoids Impending Disaster!**

Dear Editor,

We appreciate the authors for taking interest in our article and providing appropriate explanation that eluded us.^1,2^ We have tried to provide explanation to the queries raised.

A whole body computed tomography (CT) scan is done for all patients in our institute with polytrauma, however, in this case CT spine did not divulge fractures of the vertebral body. A thoracic epidural would have been contraindicated in any case of suspected spinal cord injuries too. A cervical epidural with caudal insertion of catheter was practiced by the author [SD] in the past, with a preferential distribution of injectate in the lower cervical and upper thoracic area. We agree upon the statement that although bilateral erector spinae block was eventually executed in our case, we were sceptical about the plasma levels of ropivacaine after 48th hour.

We agree that blood clots and tissue fragment might lead to a false negative aspiration and this needs to be kept in mind by anaesthesiologists who are at all levels of clinical practice. Technically, a cervical epidural needs flexion of the neck, this manoeuvre shifts the posterior dural sac anteriorly and would increase the anterior to posterior diameter of epidural space.^3^ Perhaps, this would benefit during epidural needle and a subsequent catheter placement in the cervical epidural space.

This author is accustomed to loss of resistance using air for epidural technique at all levels for more than 25 years. However, a recent Cochrane analysis mentions that identification of epidural space is driven by experience of anaesthesiologist.^4^ The article further hints at low-quality evidence that exists which do not differ between air and saline regarding loss of resistance during epidural technique considering the complications. However, this author acknowledges use of normal saline according to the recent trends.

Sandeep Diwan^1^

Abhijit Nair^2^

^1^Department of Anesthesiology, Sancheti Hospital, Maharashtra State, India

^2^Department of Anesthesiology, Ibra Hospital, Ibra-414, Sultanate of Oman

Corresponding Author: Abhijit Nair, e-mail: abhijitnair95@gmail.com
